# Low glycemic load after digestion of native starch from the indigenous tuber Belitung Taro (*Xanthosoma sagittifolium*) in a dynamic *in vitro* model of the upper GI tract (TIM-1)

**DOI:** 10.29219/fnr.v64.4623

**Published:** 2020-07-20

**Authors:** Ingrid S. Surono, Jessica Verhoeven, Koen Venema

**Affiliations:** 1 Food Technology Department, Faculty of Engineering, Bina Nusantara University, Jakarta, Indonesia; 2 Centre for Healthy Eating and Food Innovation, Maastricht University – Campus Venlo, Venlo, the Netherlands

**Keywords:** taro tuber, starch, digestibility, glycemic load, diabetes

## Abstract

**Background:**

Low glycemic foods are beneficial for people with type II diabetes. At the same time, sustained glucose release is also beneficial for people suffering from glycogen storage diseases. Taro (*Xanthosoma sagittifolium*) is a tuber indigenous to Indonesia, which has starch as the major storage carbohydrate.

**Objective:**

The aim of the current study was to determine the speed of digestion of native and modified taro starch, compared to free glucose and wheat starch.

**Design:**

This was investigated in a validated, dynamic computer-controlled *in vitro* model of the stomach and small intestine (TIM-1). Samples were taken from the dialysate, which reflected glucose absorbed in the blood stream.

**Results:**

Native taro starch showed a ~1.5-fold reduced digestibility compared to glucose and a ~ 1.35-fold compared to wheat starch. In addition, digestion of native taro starch was moved towards the ileum, and later in time compared to glucose and wheat. With modified taro starch, these effects were not observed.

**Conclusion:**

In conclusion, native taro starch showed a lower glycemic load than wheat starch and modified taro starch and could be used as a substitute for refined foods by diabetics and people suffering from other glucose metabolic diseases.

## Popular scientific summary

- Type II diabetic people have high blood glucose levels, caused by dietary ingestion of simple sugars and starch, the latter of which is digested by pancreatic amylase into glucose.
- Starch is frequently ingested from many staple foods, however not every starch is equally digestible, and hence the rise in blood glucose levels is starch-source dependent.
- Digestion of foods can be tested in a validated, sophisticated, dynamic computer-controlled in vitro model that closely mimics the stomach and small intestine of humans (TIM-1).
- Here, we show that in TIM-1 native starch from the taro-tuber is slowly digested (slower than wheat starch) and leads to sustained (low) blood glucose levels.
- Therefore, this taro-starch could be used as a substitute for refined foods by diabetics and people suffering from other glucose metabolic diseases.

The Belitung Taro *(Xanthosoma sagittifolium)* tuber, which belongs to the monocotyledonous Araceae family (the aroids), is one of the many underused indigenous tubers of Indonesia. Indonesia has the highest taro diversity in the world, and taro can be found in areas in Borneo, Java, Sumatra and Sulawesi (1, 2). To reduce dependence on rice, taro traditionally was used as an alternative carbohydrate source, but throughout Asia, it used to be an important ethnic root crop. It was cultivated and used as a staple crop in various parts of the humid tropics and sub-tropics, as it adapts well to different agro-climatic conditions (3–5).

The main (storage) carbohydrate in taro is starch. In the gastrointestinal tract (GIT), starch is degraded to glucose, maltose, maltotriose and dextrins by pancreatic amylase. Maltose, maltotriose and the dextrins are further degraded to glucose by the brush-border enzymes maltase and iso-maltase of the epithelial lining (6, 7). The speed of digestion determines blood glucose concentrations, or glycemic load (6). High glucose concentrations, amongst others due to refined food containing high amounts of glucose/sucrose and quickly digestible starch, are detrimental to diabetic individuals (8). On the other hand, sustained (slow) glucose release is beneficial for patients with, for example, glycogen storage diseases that cannot release glucose from glycogen in the liver and are therefore dependent on almost continuous glucose/starch ingestion (9). Starch can be modified to become more resistant to digestion (10). This resistant starch (RS) then reaches the colon, where it is fermented by the gut microbiota (11–13). This leads, amongst others, to the production of the health beneficial metabolite butyrate (14).

To evaluate the glycemic load, or kinetics of digestion of starch, *in vitro* models are very helpful. However, simple batch incubations usually do not predict the actual digestibility *in vivo*. Therefore, we have used here a validated, dynamic, computer-controlled *in vitro* model of the stomach and small intestine (TIM-1; [15]) with the aim to determine the glycemic load and speed of digestion of taro starch (native and after modification) compared to a free glucose solution and wheat starch. This model has been extensively validated and has been used before to determine starch digestion (6, 9, 16, 17).

## Material & methods

### Starch substrates

‘HASILBUMIKU’ taro starch was purchased from a local supplier in Bantul, Yogyakarta (Indonesia). Modified taro starch was manufactured by autoclave cooling according to a modification of the method of Zhao and Lin (10). In brief, taro starch was blended with distilled water based in a 1:3.5 ratio, and then gelatinized using pressure-heating (121°C for 30 min) and subsequent cooled at 4°C for two cycles. Afterwards, the retrograded starch was dried in an oven (60°C for 16 h) after which it was allowed to cool to room temperature for 24 h, and subsequently grinded, and sieved using a 60 mesh. Wheat flour (Jumbo basic) was purchased from Jumbo supermarket in Roermond (the Netherlands), and glucose was purchased from Merck (Darmstadt, Germany).

### Dynamic in vitro model of the stomach and small intestine (TIM-1)

**Fig. 1:**
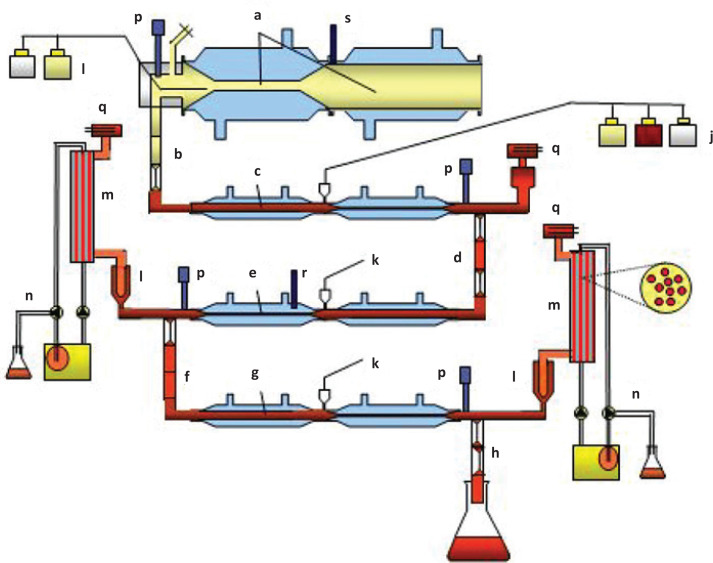
Schematic diagram of the dynamic, multi-compartmental TNO *in vitro* model of the stomach and small intestine (TIM-1). (a) Stomach compartment, (b) pyloric sphincter, (c) duodenum compartment, (d) peristaltic valve, (e) jejunum compartment, (f) peristaltic valve, (g) ileum compartment, (h) ileo-cecal sphincter, (i) stomach secretion, (j) duodenum secretion, (k) jejunum/ileum secretion, (l) pre-filter, (m) semi-permeable membrane; (n) water absorption; (p) pH electrodes; (q) level sensors; (r) temperature sensor; (s) pressure sensor. Reprinted from Keller et al. (18) with permission.

**Fig. 2:**
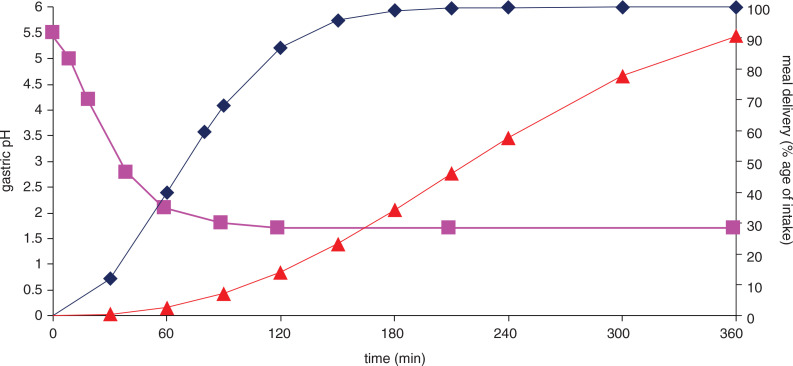
Curves mimicked in TIM-1 over time, representing the gastric (♦) and ileal delivery (▲) (both expressed as percentage of the ingested meal), and the gastric pH (■).

The dynamic model used in this study has been described in detail before (9, 16, 17). In brief, the model is composed of four serial compartments simulating stomach, duodenum, jejunum and ileum (Fig. 1). The *in vitro* digestion was performed for 6 h at 37°C. A mixture of 20 g of test-product (glucose or starch), electrolyte solution, water, pepsin (Merck, P7012), lipase (*Rhizopus* lipase, Amano Pharmaceutical Co. F-AP 15, Ltd., Nagoya Japan) and gastric start-residue were introduced into the gastric compartment. Computer-controlled peristaltic valve pumps controlled mixing through peristalsis and meal transit through the individual compartments (Fig. 2). The pH values in the compartments were computer-monitored by adding HCl (1 M; stomach) or NaHCO3 (1 M; small intestinal compartments), respectively. In the stomach, the values were preset to pH 5.5, 5.0, 4.2, 2.8, 2.1, 1.8 and 1.7 at 0, 10, 20, 40, 60, 90 and 120 min, respectively (Fig. 2). In the small intestine, the pH was maintained at 6.2, 6.5 and 7.4 in the duodenum, jejunum and ileum, respectively. Start residues were added to the compartments at the beginning of the experiment as described before (15, 16), except for the gastric start residue. Secretions of porcine bile (Merck B8631; 4 g/100 g in water) and pancreatic solution (Pancrex-V powder, Paines & Byrne, Greenford, UK; 7 g/100 g in water) entered the duodenal compartment at 0.5 and 0.25 mL/min, respectively. The absorption of digestion products and water from the jejunal and ileal compartments was simulated using hollow-fiber devices (cut-off 5–10 kDa) and samples were collected from the dialysates every hour for 6 h. These mimic the bioavailable fractions that are normally absorbed in the body, and that can be compared with blood glucose (6). Ileal efflux (indigestible fraction) was also collected every hour. At the end of the 6-h run, the residue in the system was collected to be able to create a mass balance. Experiments were performed in duplicate.

### Glucose analyses

Samples from the dialysates were quantitatively hydrolyzed to glucose with amyloglucosidase (AMG). Glucose was subsequently determined enzymatically using the Hexokinase/Glucose-6-phosphatedehydrogenase assay as described before (11). Starch and ileal efflux samples were hydrolyzed using sulfuric acid as described before (16), after which the liberated glucose was measured as described above. These analyses were performed on a Cobas Mira plus autoanalyser (Roche, Almere, the Netherlands) by Bio-aNAlytiX (Zoetermeer, The Netherlands).

## Results and discussion

Every hour samples were taken from the jejunal dialysate (Fig. 1-mn, left; Ja), ileal dialysate (Fig. 1-mn, right; Ia), and the ileal efflux (Fig. 1-h; Ie). At the end of the experiment, the residue in the system was also collected. After enzymatic (dialysates) or acid hydrolysis (ileal efflux, residue and gastric intake), free glucose was determined in these samples. Figure 1 shows the amount of glucose in the individual dialysates (Ja or Ia), as well as in the summed dialysates (Ja+Ia) for each hourly fraction as a function of recovery of the total glucose (which was >95%, data not shown).

**Fig. 3:**
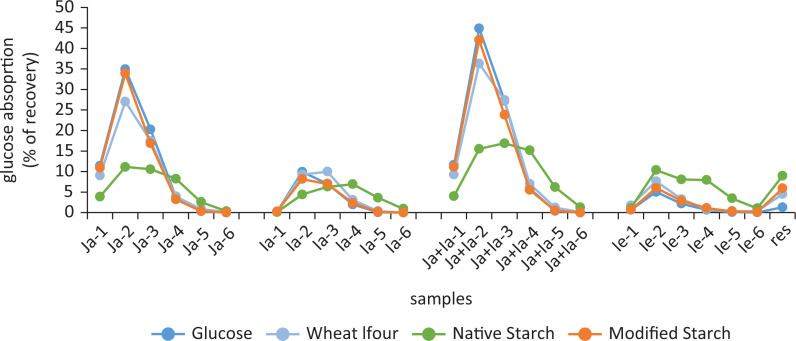
Bioaccessibility of glucose in the hourly fractions of the individual jejunal (Ja) and ileal dialysates (Ia), the summed jejunal + ileal dialysate (Ja+Ia), and ileal efflux (Ie) and residue (res) fractions.

Although glucose in solution does not require any digestion, from past experience with the model and from clinical data, we know that not all of it is absorbed. Figure 3 shows that approximately 90% is absorbed in the cumulative fractions Ja+Ia, which means that 10% is present in the Ie fractions plus the residue. Figure 3 also shows that 71% of the ingested glucose is absorbed in the jejunum (Ja; or 79% of the absorbed glucose), whereas only 19% of the ingested glucose is absorbed in the ileum (Ia; or 21% of the absorbed glucose). In total, 93% of the absorbed glucose (Ja+Ia-1 to Ja+Ia-3) becomes available in the first 3 h.

Compared to glucose, wheat starch, a highly digestible starch, follows more or less the same kinetics, but the absorbed glucose is slightly lower [81% (vs. 90% for glucose)], with 72% thereof absorbed in the jejunum and 28% in the ileum. This means that, compared to glucose, absorption is shifted a little to the ileum. Due to the lower absorption, the amount in the ileal efflux (and residue) is higher (19% vs. 10% for glucose). Also, in the first 3 h, 89% of the absorbed glucose (Ja+Ia-1 to Ja+Ia-3) becomes available for absorption, indicating that absorption is slightly slower than for free glucose, but that digestion of the wheat starch is rather efficient, since it results in very similar amounts of absorbed glucose.

Digestion of native taro starch in contrast was much lower and much slower. Overall, 59% of the ingested glucose was absorbed (37% in the jejunum; 22% in the ileum). Also, of the absorbed glucose, 62% was absorbed in the jejunum and 38% in the ileum, showing delayed absorption. Moreover, in the first 3 h, 62% of the absorbed glucose was absorbed, leaving a significant fraction to be absorbed in the last 3 h of the experiment. This can also be observed in Fig. 3, where the curve for native taro starch in the last 3 h is clearly higher than that of the other test products. Consequently, due to the low digestibility, 41% of the ingested glucose ends up in the ileal efflux (plus residue), most likely as RS, although this was not verified.

Lastly, we modified the taro starch according to a protocol by Zhao and Lin (10). They showed that RS yield with autoclaving-cooling cycles increased to 4.1% (one cycle) to 11.2% (six cycles), depending on the number of cycles. The same protocol was used for taro starch. We used three cycles, which led to an increase of ~8% by Zhao and Lin. However, in our hands, this did not lead to more RS, but resulted in a starch that was even better digestible than wheat starch (Fig. 3), with overall 83% of ingested glucose-equivalents absorbed (65% in de jejunum and 18% in the ileum). Of the absorbed glucose, 93% was absorbed in the first 3 h. Apparently, the autoclave/cooling technique does not work in the same way on different starches. After three autoclave/cooling cycles, the taro starch became better digestible upon using this treatment, rather than more resistant to digestion (data not shown).

The same model has been used for digestibility of other starch sources. For instance, maize starch was shown to be 86% digestible with a comparable protocol (9), while starch from cassava (sweet pouvilho) showed 74% digestibility (9). Similarly, two substrates designed to contain high RS, retrograded long-chain tapioca maltodextrins and high amylose maize starch, were 40.4% and 42.5% digestible, respectively (17). Native taro starch (41% resistant) was shown to have similar amounts of RS as these latter two, without any modifications.

## Conclusions

In conclusion, native taro starch is an excellent source of RS, and would lead to low and slow accumulation of glucose in blood, based on the validated *in vitro* model used here. It could be an excellent replacement for refined foods for people with type II diabetes, which are also on the rise in Indonesia, and would lead to sustained glucose release for individuals suffering from glycogen storage disease. The RS that makes it to the colon would be fermented by the gut microbiota and lead to increases in the health-beneficial microbial metabolite butyrate. This has already been shown by us in a rat model for diabetes (IS Surono et al., submitted for publication), and thus use of the indigenous tuber in the Indonesian diet holds great promise for health.

## Conflict of interest and funding

The authors declare no potential conflicts of interest. The study was funded by the Bina Nusantara Foundation with a 2019 Eminent Research Grant of Bina Nusantara University. The study was also partly funded by the Centre for Healthy Eating & Food Innovation (HEFI) of Maastricht University – campus Venlo. This research has been made possible with the support of the Dutch Province of Limburg with a grant to HEFI.
